# The Relationship Between Cardiovascular Disease Risk Score and Postoperative Delirium: The PNDABLE Study

**DOI:** 10.3389/fnagi.2022.851372

**Published:** 2022-06-21

**Authors:** Jiahan Wang, Li Wang, Xinhui Tang, Fei Wang, Siyv Liu, Xiaoyue Wu, Rui Dong, Xu Lin, Bin Wang, Yanlin Bi

**Affiliations:** ^1^Department of Anesthesiology, Qingdao Municipal Hospital Affiliated to Qingdao University, Qingdao, China; ^2^Department of Anesthesiology, Drum Tower Hospital Affiliated to Nanjing University Medical School, Nanjing, China

**Keywords:** postoperative delirium, vascular risk score, cerebrospinal fluid, biomarkers, mediation analysis

## Abstract

**Objective:**

We aimed to investigate the relationship between Framingham Heart Study general cardiovascular disease risk score (FHS–CVD risk score) and postoperative delirium (POD) among patients who had unilateral total knee arthroplasty performed under epidural anesthesia. Furthermore, we examined whether such a hypothesized relationship was mediated by the cerebrospinal fluid (CSF) biomarkers.

**Methods:**

A total of 750 participants were included in the current study. And the data were drawn from the database obtained from the Perioperative Neurocognitive Disorder And Biomarker Lifestyle (PNDABLE) study. The preoperative cognitive function of participants was measured by using Mini-Mental State Examination (MMSE). The incidence of POD was assessed using the Confusion Assessment Method (CAM). The POD severity was measured using the Memorial Delirium Assessment Scale (MDAS). The POD CSF biomarkers included in the current study were: Aβ42, T-tau, P-tau, Aβ42/T-tau, and Aβ42/P-tau. The level of the CSF biomarkers was measured using the enzyme-linked immune-sorbent assay (ELISA) in the PNDABLE study. Linear regression analysis was performed to examine the relationship between the FHS–CVD risk score and the POD CSF biomarkers. Logistic regression was used to analyze the relationship between FHS–CVD risk score, POD CSF biomarkers, and POD incidence. The proposed mediating effect of CSF biomarkers was evaluated using Mediation Analysis with 10,000 bootstrapped iterations. The receiver operating characteristic (ROC) curve is chosen as the evaluation metric for assessing the efficacy of the FHS–CVD risk score in predicting POD.

**Results:**

In the PNDABLE study, the overall incidence of POD was 22.9% with 37.2% in the higher vascular risk group and 7.9% in the lower vascular risk group. Multiple linear regression models showed that a higher preoperative FHS–CVD risk score was positively correlated with CSF T-tau (β = 0.218, *P* = 0.015) and P-tau level (β = 0.309, *P* < 0.001) in the higher vascular risk group. After adjusting for age (40–90 years), gender, education, MMSE, smoking history, drinking history, hypertension, diabetes, and the presence of CHD (cardiovascular heart disease), the results of the logistic regression analysis demonstrated the effect of Aβ42 (OR = 0.994, 95% CI 0.992–0.996, *P* < 0.001), Aβ42/T-tau (OR = 0.353, 95% CI 0.254–0.491, *P* < 0.001), and Aβ42/P-tau (OR = 0.744, 95% CI 0.684–0.809, *P* < 0.001) in protecting patients against POD. However, the FHS–CVD risk score (OR = 1.142, 95% CI 1.017–1.282, *P* = 0.025) and the remaining two biomarkers: T-tau (OR = 1.005, 95% CI 1.004–1.007, *P* < 0.001) and P-tau (OR = 1.045, 95%CI 1.029–1.062, *P* < 0.001) were identified as the risk factors. Mediation analyses revealed that the association between FHS–CVD risk score and POD was partially mediated by T-tau (proportion: 31.6%) and P-tau (proportion: 23.6%). The predictive power of the FHS–CVD risk score was validated by the ROC curve with an AUC of 0.7364.

**Conclusion:**

Higher vascular risk score is one of the preoperative risk factors for POD, which is partly mediated by CSF biomarker tau protein.

**Clinical Trial Registration:**

[www.clinicaltrials.gov], identifier [ChiCTR2000033439].

## Introduction

Postoperative delirium (POD), an acute or fluctuating mental status impairment, is one of the common adverse postoperative complications and the elderly are at higher risk because the predisposing risk factors are often associated with aging (Blazer and van Nieuwenhuizen, 2012). With the rapid aging of the population, the number of people with dementia is increasing (Young and Inouye, 2007; Aldecoa et al., 2017; Jia et al., 2020). The characteristics are perceptual, awareness, attention, and cognition dysfunction (Monk and Price, 2011; Blazer and van Nieuwenhuizen, 2012). POD starts during the recovery room and often occurs in the hospital up to 1-week post procedure or until discharge (Evered et al., 2018). The occurrence of delirium not only increases the patient’s economic burden, prolonging the hospital stay, but also easily leads to the reduction of quality, leading to the inability to live normally (Witlox et al., 2010). However, there are no effective measures to prevent or cure dementia. Hence, it is crucial to identify the pathogenesis mechanisms and to find out the potential risk factors.

The cognitive impairment has been confirmed in connection with the multiple vascular and environmental risk factors. After fully validated and integrated multivariate variables, the Framingham Heart Study (FHS) was aimed at searching for the risk factors of cardiovascular disease (CVD) and predicting the 10-year risk of CVD to improve the prevention by formulating health guidelines (Vemuri et al., 2017). Over several decades, FHS has expanded to be an almost comprehensive study and grown into a three-generational prospective community-based cohort study of dementia and Alzheimer’s disease (AD) and of the preclinical states preceding AD including various types of mild cognitive impairment (MCI) and pre-MCI (Dawber and Kannel, 1966; Feinleib et al., 1975; Splansky et al., 2007). Correlational studies have proven that FHS–CVD risk score is more applicable to assess the risk of cognitive decline.

Postoperative delirium is one of the most common postoperative neurodegenerative changes. Plenty of evidences suggest that cerebrospinal fluid (CSF) amyloid β 42 (Aβ42), total tau (T-tau), and phospho-tau (P-tau) are biomarkers of the POD process, reflecting plaque pathology, neurodegeneration, and neurofibrillary tangle pathology, respectively (Maldonado, 2013). The aforementioned biomarkers can be seen before the clinical emergence of dementia (Sperling et al., 2011). Lower preoperative CSF Aβ level and higher tau level are associated with both higher incidence and greater symptom severity of POD, which can distinguish patients with dementia from the healthy controls and predict the development of it. These results suggest that changes in the levels of Aβ and tau in CSF may be a common neuropathogenesis underlying both postoperative delirium and postoperative cognitive dysfunction.

To date, there has been no research demonstrating the overall impact of multiple vascular risk factors on cognitive function. Therefore, the purpose of this study is to explore the relationship between FHS–CVD risk score and POD, and to test whether the influences of FHS–CVD risk score on POD were mediated by the POD biomarkers.

## Materials and Methods

### Perioperative Neurocognitive Disorder and Biomarker LifestylE Study

The Perioperative Neurocognitive Disorder and Biomarker LifestylE study (PNDABLE) aims at investigating the pathogenesis, risk factors, and biomarkers of perioperative neurocognitive disorders in the northern Chinese Han population. The purpose of PNDABLE is to identify lifestyle factors that may affect the risk of PND in the non-demented northern Chinese Han population to provide a foundation for disease prevention and early diagnosis. This study has been registered in the Chinese Clinical Trial Registry (clinical registration number ChiCTR2000033439) and approved by the Ethics Committee of Qingdao Municipal Hospital. CSF samples could be collected from all the participants after the written informed consent was obtained from the patients or their legal representatives.

### Participants

The Han Chinese patients undergoing unilateral total knee arthroplasty (no gender limitations, aged 40 ∼ 90, ASA I ∼ II) combined with epidural anesthesia were enrolled in the PNDABLE study Qingdao Municipal Hospital. The exclusion criteria include: (1) preoperative MMSE score < 24 points; (2) drug or psychotropic substance abuse, as well as long-term use of steroid drugs and hormone drugs; (3) pre-operative III-IV hepatic encephalopathy; (4) recent major surgery; (5) severe visual and hearing impairments; (6) abnormal coagulation function before surgery; (7) central nervous system infection, head trauma, multiple sclerosis, neurodegenerative diseases other than AD (e.g., epilepsy, Parkinson’s Disease), or other major neurological disorders; (8) major psychological disorders; (9) severe systemic diseases (e.g., malignant tumors) that may affect CSF or blood levels of AD biomarkers including Aβ and tau; and (10) family history of genetic diseases.

**FIGURE 1:**
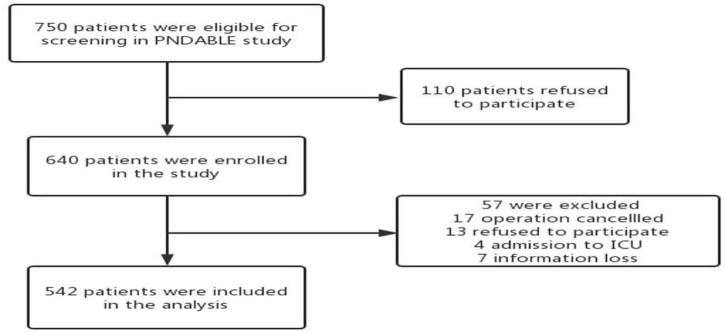
The flow diagram showed that 750 participants were initially screened for the PNDABLE studies and finally 542 participants were included in the data analysis.

We collected a total of 750 cognitively normal participants’ available information on covariates from the PNDABLE study. According to whether POD occurred or not, all the participants were divided into two groups: POD group and NPOD group. A patient recruitment flowchart is shown in Figure 1.

The participants did not receive preoperative medications, and they were instructed not to drink for 6 h and not to eat for 8 h before surgery. After entering the operating room, we routinely monitored ECG, SpO_2_, and NBP, opened vein access and extracted 3 ml of whole venous blood. All the patients underwent a combined spinal–epidural block, with the space between lumbar 3-4 spinous processes (L3-4) as the puncture site. After a successful puncture, 2 ml of cerebrospinal fluid was extracted from the subarachnoid space, followed by injection of 2–2.5 ml ropivacaine (0.66%) for about 30 s. After anesthesia, the sensory level was controlled below the T8 level. During the surgery, oxygen was inhaled *via* a mask at 5 L/min to maintain blood pressure within ± 20% of the baseline value. If intraoperative NBP < 90 mmHg (1 mmHg = 0.133 kPa) or it decreased by more than 20% of the baseline value, ephedrine 5 mg was injected intravenously. If HR < 50 beats/min, atropine 0.5 mg was injected intravenously. Intravenous patient-controlled analgesia (butorphanol 0.1 mg/ml + tropisetron 0.05 mg/ml, diluted with normal saline to a total volume of 100 ml) was used in acute postoperative pain management. After the operation, the patient was sent to the anesthesia resuscitation room (PACU).

We interviewed all the patients the day before surgery and collected their baseline data, including age, gender, body mass index (BMI), ASA physical status, and years of education. Other information including comorbidities, medical history, and fracture classifications were also collected according to the patients’ medical records. The whole history collection, physical examination, and dementia-related cognitive assessment were conducted by an anesthesiologist.

### Cerebrospinal Fluid Biomarkers of Postoperative Delirium Measurements

Cerebrospinal fluid samples were processed immediately within 2 h after standard lumbar puncture. Each sample was centrifuged at 2,000 × g for ten minutes, and CSF samples were separated and stored in an enzyme-free EP (Eppendorf) tube (AXYGEN; PCR-02-C) at −80°C under the international BIOMARKAPD project for further use in the subsequent steps of this study.

Cerebrospinal fluid biomarkers of POD were measured by ELISA using the microplate reader (Thermo Scientific Multiskan MK3). CSF biomarkers of POD measurements were done with other ELISA kits [Aβ42 (BioVendor, Ghent, Belgium Lot: No.296-64401), P-tau (BioVendor, Ghent, Belgium Lot: QY-PF9092) and T-tau (BioVendor, Ghent, Belgium Lot: No. EK-H12242)]. All the ELISA measurements were performed by experienced technicians in strict accordance with the manufacturer’s instructions. They knew nothing about the clinical information. The samples and standards were measured in duplicates, and the means of duplicates were used for the statistical analyses. All the antibodies and plates were from a single lot to exclude variability between batches. Moreover, the within-batch CV was < 5% and the inter-batch CV was < 15%.

### Assessment Criteria for Framingham Heart Study–Cardiovascular Disease Risk Score

The FHS–CVD risk score is a risk forecasting tool, which highlights gender specification and summarizes multiple factors to assess vascular accident within 10 years. The FHS–CVD risk score is calculated synthetically based on the participants’ age, gender, antihypertensive therapy condition, systolic pressure, total cholesterol, high-density lipoprotein, smoking history, and diabetes history. The higher the patient’s vascular risk score, the more severely the patient’s vessels have aged, suggesting the higher risk of future vascular events. Previous studies have confirmed that individuals with a calculated 10-year risk of CAD-related death or non-fatal MI of 20% or greater are considered to be in the ‘high-risk’ category (McPherson et al., 2006).

### Neuropsychological Tests

The preoperative cognitive status of participants was assessed by neurologists using the Mini-Mental State Examination (MMSE) assessed. Patients whose MMSE scores < 24 points were excluded.

The delirium assessment was performed at 9:00–10:00 a.m. and 2:00–3:00 p.m. twice a day on 1–7 days (or before discharge) by an anesthesiologist postoperatively. We used the visual analog scale (VAS) score of 0–10 (lower scores indicating lower levels of pain) to assess pain at the same time. POD was defined by the Confusion Assessment Method (CAM), and POD severity was measured by using the Memorial Delirium Assessment Scale (MDAS). Therefore, CAM-positive and MDAS-positive patients postoperatively were recorded.

### Statistical Analysis

The preliminary test in this study found that 6 covariates were expected to enter the Logistic regression, the POD incidence was 10%, and the loss of follow-up rate was assumed to be 20%, so the required sample size was calculated to be 750 cases (6 × 10÷0.1÷0.8 = 750).

The Kolmogorov–Smirnov test was used to determine whether the measurement data conformed to the normal distribution. The measurement data conformed to the normal distribution was expressed by mean ± standard deviation (SD), while the median (p25, p75) or a number (%) to express the data. We used × 2 tests (for categorical variables) and Mann–Whitney *U* test (for continuous variables) to test the difference of baseline characteristics between higher vascular risk group and lower vascular risk group. And the differences of CSF biomarkers levels between the higher and lower vascular risk groups were described in Box plots.

At first, we tested the relationships of the FHS–CVD risk scores with CSF POD biomarkers. Linear regression analysis of FHS–CVD risk score and Aβ42, T-tau, P-tau, Aβ42/T-tau, and Aβ42/P-tau was performed in the higher and lower vascular risk group, respectively. We also tested the relationships of POD with CSF POD biomarkers and FHS–CVD risk score. Binary logistic regression analysis of POD and FHS–CVD risk score, Aβ42, T-tau, P-tau, Aβ42/T-tau, and Aβ42/P-tau was performed.

Second, sensitivity analyses were performed by: (1) we tested the relationships of POD with FHS–CVD risk score and CSF POD biomarkers for patients adding four covariates, including age of 40∼90 age, gender, years of education and MMSE in multivariate logistic regression analysis; (2) we also tested the relationships of POD with FHS–CVD risk score and CSF POD biomarkers for patients over the age of 40∼90 age, gender, years of education and MMSE adding more covariates, including hypertension (yes or no), type 2 diabetes (yes or no), coronary heart disease (yes or no), smoking (yes or no), and alcohol intake (yes or no) were chosen as covariates in the multivariate logistic regression analysis.

Third, to examine whether the association between FHS–CVD risk score and POD was mediated by CSF POD biomarkers, linear regression models were fitted based on the methods proposed by Baron and Kenny (31). The first equation regressed the mediator (CSF POD biomarkers) on the independent variable (FHS–CVD risk score). The second equation regressed the dependent variable (POD) on the independent variable. The third equation regressed the dependent variable on both the independent variable and the mediator variable. Mediation effects were established if the following criteria were simultaneously reached: (1) FHS–CVD risk score was significantly related to CSF POD biomarkers; (2) FHS–CVD risk score was significantly related to POD; (3) CSF POD biomarkers were significantly related to POD; and (4) the association between FHS–CVD risk score and POD was attenuated when CSF POD biomarkers (the mediator) were added in the regression model. Furthermore, the attenuation or indirect effect was estimated, with the significance determined using 10,000 bootstrapped iterations, where each path of the model was controlled for age, sex, education, and MMSE.

Finally, the effectiveness of the FHS–CVD risk score in predicting POD was evaluated by introducing area under curve (AUC) of ROC curves.

The data were analyzed by using the Stata MP16.0 (Solvusoft Corporation, Inc., Chicago, IL, United States), GraphPad Prism version 8.0 (GraphPad Software, Inc., LaJolla, CA, United States), and R software version 4.4.1(R Foundation for Statistical Computing, Vienna, Austria).

## Results

### Participant Characteristics

**FIGURE 2:**
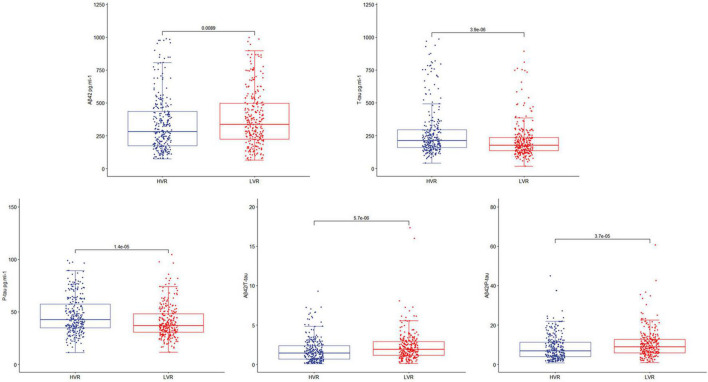
The box-plots showed the level of CSF POD biomarkers in the higher vascular risk group (HVR) and the lower vascular risk group (LVR). The result showed that CSF T-tau and P-tau levels of the participants were higher in the higher vascular risk group.

This study enrolled 750 participants. In total, five hundred forty-two (*n* = 542) were eligible for analysis, and 208 participants were excluded. The criteria for exclusion are shown in Figure 1. The demographical and clinical data are summarized in Table 1. The incidence of POD was observed at 22.9% (*n* = 124/542), with 37.2% (*n* = 103/277) in the higher vascular risk group and 7.9% (*n* = 21/265) in the lower vascular risk group. There was a statistically significant difference in POD occurrence between two groups (*P* < 0.05). And the POD CSF T-tau and P-tau levels were higher in the higher vascular risk group (Figure 2).

**TABLE 1:** Demographic and clinical characteristics of participants.

Variable	Higher vascular risk group	Lower vascular risk group	*P* value
Age, yr	68.0(63.0,74.0)	56.0(49.0,62.0)	< 0.001
Female	50(18.1)	156(59.1)	< 0.001
Education, yr	9.0(9.0,12.0)	12.0(9.0,12.0)	0.032
CM-MMSE	28.0(26.5,29.0)	29.0(27.0,30.0)	< 0.001
MDAS	3.0(1.0,9.0)	1.0(1.0,8.0)	< 0.001
Hypertension	109(39.4)	78(29.4)	0.015
Diabetes	48(17.3)	42(15.8)	0.644
CHD	34(12.3)	40(15.1)	0.339
Smoking history	124(44.8)	34(12.8)	< 0.001
Drinking history	97(35.0)	76(28.7)	0.114
FHS-CVD risk score	18.0(16.0,19.0)	14.0(12.0,19.0)	< 0.001
Aβ42,pg⋅ml^–1^	281.6(174.3,435.1)	335.5(222.9,498.8)	0.009
T-tau,pg⋅ml^–1^	211.1(159.6,296.0)	176.9(134.4,236.5)	< 0.001
P-tau,pg⋅ml^–1^	42.5(34.7,58.0)	37.1(30.6,48.3)	< 0.001
Aβ42/T-tau	1.5(0.7,2.4)	1.9(1.0,2.9)	< 0.001
Aβ42/P-tau	6.8(3.9,11.3)	8.9(5.9,12.8)	< 0.001
POD occurrence	103 (37.2)	21(7.9)	< 0.001

*Continuous variable use Student’s t test or Mann-Whitney U, Categorical variable use Chi-square test.*

*The numerical variables of normal distribution are statistically described by Average Standard deviation.*

*Non-normally distributed numerical variables are statistically described by Interquartile range(IQR).*

*Categorical variables are statistically described by Sample size, Percent.*

### The Relationship Between Framingham Heart Study–Cardiovascular Disease Risk Score and Cerebrospinal Fluid Postoperative Delirium Biomarkers

**FIGURE 3:**
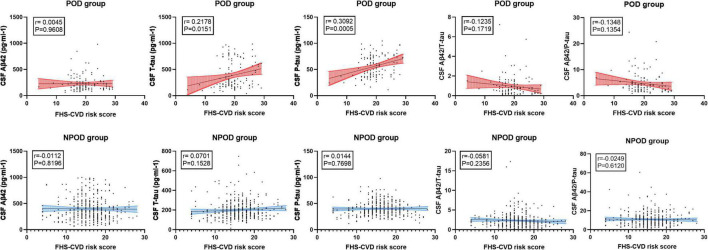
The linear regression models showed the relationship between the CSF POD biomarkers and FHS–CVD risk score. The result showed that higher preoperative FHS–CVD risk score were positively correlated with CSF T-tau and P-tau level in the POD group.

The concentrations of CSF biomarkers (Aβ42, T-tau, P-tau, Aβ42/T-tau, and Aβ42/P-tau) before operation in the higher and lower vascular risk group were compared with FHS–CVD risk score to investigate the relationship in linear regression analysis. Multiple linear regression models showed that higher preoperative FHS–CVD risk score were positively correlated with CSF T-tau (β = 0.218, *P* = 0.015) and P-tau level (β = 0.309, *P* < 0.001), while were not correlated with CSF Aβ42 (β = 0.004, *P* = 0.961), Aβ42/T-tau (β = −0.123, *P* = 0.172) and Aβ42/P-tau level (β = −0.135, *P* = 0.135) in the POD group. However, multiple linear regression models showed that higher preoperative FHS–CVD risk score were not correlated with CSF Aβ42 (β = −0.011, *P* = 0.820), T-tau (β = 0.070, *P* = 0.153), P-tau (β = 0.014, *P* = 0.770), Aβ42/T-tau (β = −0.058, *P* = 0.236), and Aβ42/P-tau level (β = −0.025, *P* = 0.612) in the NPOD group. And the linear regression results of the connection between FHS–CVD risk score and CSF POD biomarkers were shown in Figure 3.

### The Relationship Among Framingham Heart Study–Cardiovascular Disease Risk Score, Cerebrospinal Fluid Biomarkers, and Postoperative Delirium

We compared the concentrations of CSF biomarkers (Aβ42, T-tau, P-tau, Aβ42/T-tau, and Aβ42/P-tau) and FHS–CVD risk score between patients with POD and non-POD before operation. Binary logistic regression analysis showed that the level of Aβ42(OR = 0.994, 95% CI = 0.992–0.995, *P* < 0.001), Aβ42/T-tau(OR = 0.285, 95% CI = 0.211–0.384, *P* < 0.001), and Aβ42/P-tau (OR = 0.714, 95% CI = 0.661–0.770, *P* < 0.001) maintained great significance and were protective factors of POD, while T-tau OR = 1.006, 95% CI = 1.005–1.008, *P* < 0.001), P-tau (OR = 1.059, 95% CI = 1.045–1.073, *P* < 0.001) and FHS–CVD risk score (OR = 1.231, 95% CI = 1.165–1.300, *P* < 0.001)were risk factors of POD (Table 2). To verify the stability of the results, we performed sensitivity analyses, respectively, using two models based on more covariables on studies. FHS–CVD risk score, Aβ42, T-tau, P-tau, Aβ42/T-tau, and Aβ42/P-tau remained stable across two sensitivity analyses (Table 2). To sum up, the sensitivity analysis has showed that the results were stable.

**TABLE 2:** The logistic regression analysis and sensitivity analysis among FHS-CVD risk score, CSF POD biomarkers and POD.

	Unadjusted		Adjusted1		Adjusted2	
	OR(95%CI)	*P* value	OR(95%CI)	*P* value	OR(95%CI)	*P* value
Aβ42,pg⋅ml^–1^	0.994(0.992−0.995)	< 0.001	0.994(0.992−0.996)	< 0.001	0.994(0.992−0.996)	< 0.001
T-tau,pg⋅ml^–1^	1.006(1.005−1.008)	< 0.001	1.005(1.003−1.007)	< 0.001	1.005(1.004−1.007)	< 0.001
P-tau,pg⋅ml^–1^	1.059(1.045−1.073)	< 0.001	1.045(1.029−1.061)	< 0.001	1.045(1.029−1.062)	< 0.001
Aβ42/T-tau	0.285(0.211−0.384)	< 0.001	0.362(0.262−0.501)	< 0.001	0.353(0.254−0.491)	< 0.001
Aβ42/P-tau	0.714(0.661−0.770)	< 0.001	0.746(0.687−0.811)	< 0.001	0.744(0.684−0.809)	< 0.001
FHS-CVD risk score	1.231(1.165−1.300)	< 0.001	1.148(1.026−1.283)	0.016	1.142(1.017−1.282)	0.025

*Adjusted1: adjusted for age (40−90), gender, education and MMSE.*

*Adjusted2: adjusted for age (40−90), gender, education, MMSE, smoking history, drinking history, hypertension, diabetes and CHD.*

### Causal Mediation Analyses

**FIGURE 4:**
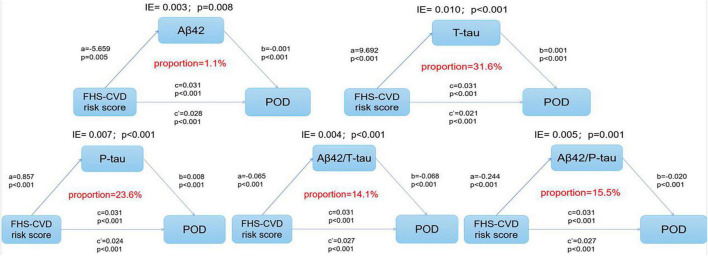
The mediation analysis showed that the relationship between FHS–CVD risk score and POD was mediated by T-tau and P-tau protein.

The multivariate regression in PNDABLE study showed that FHS–CVD risk score, T-tau, and P-tau were positively correlated with POD, while Aβ42, Aβ42/T-tau, and Aβ42/P-tau were negatively correlated with POD. Therefore, we speculated that FHS–CVD risk score is not only a risk factor of POD but may also regulate the occurrence of POD through Tau pathology. We further explored whether T-tau and P-tau could mediate the effect of FHS–CVD risk score on POD. The mediation analysis showed that the relationship between FHS–CVD risk score and POD was mediated by T-tau (the proportion of intermediaries is about 31.6%) and P-tau (the proportion of intermediaries is about 23.6%) (Figure 4). As the result showed, T-tau accounted for the largest proportion of the effect of vascular risk factors on the occurrence of POD among the CSF biomarkers.

### Receiver Operating Characteristic Curve

**FIGURE 5:**
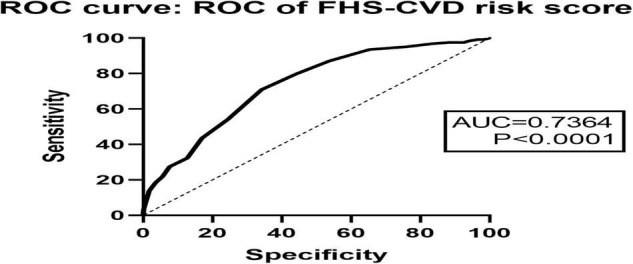
The ROC curve showed that FHS–CVD risk score had effective diagnostic significance in predicting POD occurrence.

The ROC curve showed that the FHS–CVD risk score (AUC = 0.7364) was effective in predicting POD occurrence (Figure 5).

## Discussion

After analyzing 542 participants under combined spinal–epidural anesthesia in this study, we found that FHS–CVD risk score is one of the preoperative risk factors for POD. The person with higher vascular risk scores had worse postoperative cognitive functioning overall. The underlying mechanism of such a relationship appears to be correlated with the significant association between the FHS–CVD risk score and tau protein deposition in cerebrospinal fluid. Our findings highlight the important role of adverse vascular risk in the pathogenesis of POD and postoperative cognitive decline.

Postoperative delirium is a syndrome associated with the interaction of patients’ demographic factors, basic disease factors, and anesthesia and surgery factors (Aldecoa et al., 2017). As the most common postoperative neurodegenerative change, the incidence rate of POD is climbing with the arrival of global aging. However, there is currently a lack of effective treatments to treat POD, and it becomes a serious problem affecting postoperative prognosis. In recent years, epidemiological studies have played an important role in cognitive prevention of POD. A large number of studies have centralized and summarized potential controllable factors that can significantly reduce the prevalence of POD in asymptomatic preclinical stages. Due to its vital role in maintaining adequate blood supply and healthy substance exchange, vascular dysfunction is receiving increasing attention in the pathogenesis of POD (Iadecola, 2017).

Previous studies have confirmed that a variety of vascular risk factors will increase the risk of dementia (Hofman et al., 1997). Pathological injury and vascular injury coexist and then act together to promote the progression of POD (Regan et al., 2006; Tariq and Barber, 2018). After the well control of vascular risk factors, the prevalence of cognitive dysfunction has decreased in many developed countries (Hinterberger et al., 2016). However, there has no single vascular risk factor has been shown the associated with the incidence and development of the POD (Iadecola, 2013; He et al., 2020). Considering the interaction of the vascular risk factors, our study decides to summarize the influence of vascular risk factors to get the potential reliable conclusion.

The concentration of CSF biomarkers such as Aβ42, T-tau, P-tau, Aβ42/T-tau, and Aβ42/P-tau in patients have been used as a set of important indicators in previous clinical research and POD diagnosis (McKhann et al., 2011; Olsson et al., 2016; Cunningham et al., 2019). Specifically, increases in the T-tau level and P-tau level, respectively, reflect the intensity of axial degeneration and the amount of neurofibrillary tangles detected in patients with brain pathology. Regarding the amyloid protein Aβ42, its accumulation in amyloid plaques during POD pathology changes was shown to decrease the concentration of Aβ42 in CSF (Cunningham et al., 2019). Since the concentration of POD biomarkers have changed in the preclinical stage, there is an opportunity to investigate the relationship between the vascular risk factors and POD pathological changes.

According to our findings, we found that patients with higher vascular risk are often associated with higher CSF T-tau and P-tau levels, yet their CSF Aβ42, Aβ42/T-tau, and Aβ42/P-tau levels appeared to be lower. The results showed that there were significant differences in the expression of Aβ42, T-tau, P-tau, Aβ42/T-tau, and Aβ42/P-tau in CSF between higher vascular risk group and lower vascular risk group (*P* < 0.05), indicating an increased risk of POD in patients with higher vascular risk. Although there exist a few hypotheses about the underlying mechanism of the observed association between vascular risk factors and cognitive dysfunction, how one factor interacts with or perhaps causes the others remains unclear. So far, increasing evidence has been found for the cerebral hypoperfusion hypothesis, which postulates that the increased risk of vascular sclerosis and infraction could impair the cerebral vascular automatic regulation function and decrease intracranial blood supply. Chronic cerebral hypoperfusion leads to pathological changes such as abnormal protein synthesis, disturbance of energy metabolism, decreased glucose utilization, and loss of choline receptors in brain regions, and further leads to loss of neurons and impaired function (de la Torre, 2000, 2002). And similar pathological features can be found in brain ischemic diseases and POD like amyloid and tau protein changes, which is consistent with our findings.

As the mediation effect analysis showed, T-tau accounted for the largest proportion of the effect of vascular risk factors on the occurrence of POD among the POD CSF biomarkers. Previous studies have shown that vascular risk factors contribute to increased tau deposition (Vemuri et al., 2017). Studies have also found that cerebral ischemia itself can express hyperphosphorylated tau protein, which suggests that cerebral ischemia can promote POD pathological changes (Iadecola, 2013). The activation of the cell cycle signal transduction system was induced by cerebral ischemic injury, and cells enter into abnormal mitosis and apoptosis. This could lead to hyperphosphorylation of tau protein and formation of nerve fiber tangles, thus, participating in the pathological progress of POD (Stadelmann et al., 1999; Kang et al., 2013; Ye et al., 2020). So far, no studies have proven a correlation between FHS–CVD risk score and CSF Aβ42 level (Gupta et al., 2015). Nonetheless, there is evidence that brain ischemia induces ischemic generation of amyloid plaques, which can interact with vascular changes in the brain and progress to POD (Vemuri et al., 2017).

The clear and reliable relationship between the single vascular risk factor and POD incidence has not been shown in the previous studies. However, studies have shown that long-term hypertension causes changes in cerebral blood flow and brain atrophy, resulting in neuronal damage and other pathological changes, which can cause cognitive disorders (Whitmer et al., 2005). The relative or absolute lack of insulin in the early stage of insulin resistance and type II diabetes can cause cognitive decline, which share common neuropathological characteristics (Hughes and Craft, 2016). Smoking would exacerbate the risk of vascular disease, chronic inflammation, and oxidative stress, all of which are potential causes of cognitive dysfunction (Anstey et al., 2007). As an important agent of Aβ protein clearance through the blood–brain barrier, low-density lipoprotein receptor-related protein 1(LRP1) may be used as a therapeutic target for regulating the pathological changes of cognitive disorders (Sparks et al., 2006; Storck and Pietrzik, 2017).

It should also be emphasized that it is a group of vascular risk factors but not a single one, that may ultimately lead to vascular dysfunction. FHS–CVD risk score is a simple but reliable vascular risk assessment tool, which could be used by considering multiple vascular risk and disadvantages conditions simultaneously. Visual inspection of the ROC curve, our research finding indicated that FHS–CVD risk score was accurate in predicting POD. The items evaluated by the FHS–CVD risk score are mostly curable. Together with the comprehensive range of items included, such an assessment tool could be used as a valuable guidance for the clinicians to design their prevention and treatment plan. The study has potential implications for public health, and good use of medications such as antihypertensive, hypoglycemic, and lipid-lowering drugs may prevent, slow, or even reverse the progression of postoperative delirium (Sparks et al., 2006; Duron and Hanon, 2008; Vizcaychipi et al., 2014). The public is called on for active primary or secondary prevention.

The limitations of our study are as follows: first, according to the data from the PNDABLE, we could only assess the association among the FHS–CVD risk score, POD biomarkers, and POD incidence in the cross-sectional study. We are able to improve the longitudinal data on patients in the future study, and further clarify the association. Second, this study only focused on the relationship between vascular risk factors of the FHS–CVD risk score and the CSF biomarkers of POD and did not involve other related pathogenesis of POD, which may influence the POD incidence. AT third, this study was carried out among hospitalized patients, who are scheduled for surgery. The conclusion remains to be validated in animal research for further research.

## Conclusion

To sum up, adverse vascular risk burden is one of the preoperative risk factors for promoting POD. The patient with higher FHS–CVD risk score has the higher occurrence of POD. And our study found the relationship between FHS–CVD risk score and POD might be partly mediated by CSF tau protein. Early prevention of POD can improve patient quality of life, shorten the hospital stays, and reduce the burden on family and society.

## Data Availability Statement

The raw data supporting the conclusions of this article will be made available by the authors, without undue reservation.

## Ethics Statement

The studies involving human participants were reviewed and approved by Chinese Clinical Trial Registry (clinical registration number ChiCTR2000033439) and approved by the Ethics Committee of Qingdao Municipal Hospital. The patients/participants provided their written informed consent to participate in this study.

## Author Contributions

YB conceived the current study. XT, FW, SL, and XW performed the experiments. RD, XL, LW, and BW analyzed the data. JW and BW performed the experiments, wrote, and revised the manuscript. All authors have contributed to the manuscript revising and editing critically for important intellectual content and given final approval of the version and agreed to be accountable for all aspects of the work presented here.

## Conflict of Interest

The authors declare that the research was conducted in the absence of any commercial or financial relationships that could be construed as a potential conflict of interest.

## Publisher’s Note

All claims expressed in this article are solely those of the authors and do not necessarily represent those of their affiliated organizations, or those of the publisher, the editors and the reviewers. Any product that may be evaluated in this article, or claim that may be made by its manufacturer, is not guaranteed or endorsed by the publisher.
